# Small-Molecule Inhibitors of Chikungunya Virus: Mechanisms of Action and Antiviral Drug Resistance

**DOI:** 10.1128/AAC.01788-20

**Published:** 2020-11-17

**Authors:** Kristina Kovacikova, Martijn J. van Hemert

**Affiliations:** a Department of Medical Microbiology, Leiden University Medical Center, Leiden, The Netherlands

**Keywords:** direct-acting antivirals, host-directed antivirals, drug resistance, viral target, high-throughput screening, enzymatic assays, *in silico* screening, *in vivo* validation, chikungunya virus

## Abstract

Chikungunya virus (CHIKV) is a mosquito-transmitted alphavirus that has spread to more than 60 countries worldwide. CHIKV infection leads to a febrile illness known as chikungunya fever (CHIKF), which is characterized by long-lasting and debilitating joint and muscle pain. CHIKV can cause large-scale epidemics with high attack rates, which substantiates the need for development of effective therapeutics suitable for outbreak containment. In this review, we highlight the different strategies used for developing CHIKV small-molecule inhibitors, ranging from high-throughput cell-based screening to *in silico* screens and enzymatic assays with purified viral proteins. We further discuss the current status of the most promising molecules, including *in vitro* and *in vivo* findings. In particular, we focus on describing host and/or viral targets, mode of action, and mechanisms of antiviral drug resistance and associated mutations. Knowledge of the key molecular determinants of drug resistance will aid selection of the most promising antiviral agent(s) for clinical use. For these reasons, we also summarize the available information about drug-resistant phenotypes in *Aedes* mosquito vectors. From this review, it is evident that more of the active molecules need to be evaluated in preclinical and clinical models to address the current lack of antiviral treatment for CHIKF.

## Abstract (precis)

Chikungunya virus (CHIKV) is a mosquito-transmitted alphavirus that has spread to more than 60 countries worldwide. CHIKV infection leads to a febrile illness known as chikungunya fever (CHIKF), which is characterized by long-lasting and debilitating joint and muscle pain. CHIKV can cause large-scale epidemics with high attack rates, which substantiates the need for development of effective therapeutics suitable for outbreak containment. In this review, we highlight the different strategies used for developing CHIKV small-molecule inhibitors, ranging from high-throughput cell-based screening to *in silico* screens and enzymatic assays with purified viral proteins.

## INTRODUCTION

Chikungunya virus (CHIKV) is a mosquito-borne alphavirus belonging to the *Togaviridae* family that can cause explosive epidemics of acute and chronic arthritis in humans. The main vectors responsible for its transmission are the day-biting Aedes aegypti and Aedes albopictus mosquitoes. CHIKV was first isolated from a febrile patient in 1952/1953 in what is currently Tanzania (1). In the following years, it caused periodic local outbreaks in Africa and Asia. In 2004, CHIKV reemerged in coastal Kenya (2), from which it spread to immunologically naive populations in La Reunion Island and in surrounding Indian Ocean islands and South Asia during 2005 to 2006. During this outbreak, a new CHIKV variant harboring the A226V amino acid substitution in the E1 glycoprotein was isolated (3), and that isolate was more efficiently transmitted by the Aedes albopictus mosquitoes that are abundant in the temperate regions of the Americas, Europe, and Africa than earlier isolates. In late 2013, CHIKV caused the first locally transmitted outbreak on the Caribbean island of St Martin (4), resulting in more than 2.5 million cases across Central and South America in the period between 2014 and 2017 (https://www.paho.org/hq/index.php?option=com_topics&view=rdmore&cid=5927&Itemid=40931&lang=en). In Europe, the first autochthonous outbreak was described in Italy in 2007 (5) and since then renewed CHIKV transmission has occurred in Italy in 2017 (6) and southern France in 2010, 2014, and 2017 (7–9).

Chikungunya fever (CHIKF) typically begins with a sudden onset of fever 3 to 7 days after a bite of an infected mosquito, followed by symptoms such as rash, myalgia, and polyarthralgia. Polyarthralgia is mainly symmetrical and peripheral, affecting the small joints of wrists, ankles, and phalanges, as well as the larger joints such as the knee and the elbow (10). The patients usually report incapacitating pain that can last for weeks to months. CHIKF treatment has been focused entirely on relieving patients’ symptoms with analgesics, antipyretics, and anti-inflammatory agents. Nevertheless, some of these drugs can have serious side effects upon prolonged use. The current lack of clinically approved therapeutics and adequate control measures for CHIKF warrants the development of safe and effective antiviral therapy.

In this review, a comprehensive overview of small-molecule inhibitors of CHIKV is presented in Tables 1 to 4 grouped by the approach by which they were identified. Tables 1 and 2 list inhibitors that were identified by cell-based screening, Table 3 lists compounds identified by *in silico* approaches, and Table 4 lists compounds that were identified in enzymatic assays. Note that the values presented in Tables 1 to 4 are not directly comparable as the experimental parameters/setups differed between studies, e.g., the use of different virus isolates, multiplicities of infection (MOIs), readouts (cytopathic effect [CPE] versus titers versus quantitative real-time PCR [qRT-PCR]), times of harvest, and types of mouse models. Since this review focuses on small-molecule inhibitors, we have not included small interfering RNA (siRNA)-mediated gene silencing approaches.

**TABLE 1:** Compounds targeting CHIKV entry and egress^*a*^

Compound^*b*^	Viral target	Resistance mutation(s)	*In vitro* efficacy				*In vivo* efficacy			Reference(s)
			CHIKV strain (genotype)^*c*^	EC_50_ (μM) or other readout^*d*^	CC_50_ (μM)	Cell line	CHIKV strain (genotype)	Efficacy	Mouse model	
Obatoclax (R)	E1	L369I (SFV)	LR2006 OPY1 (ECSA genotype)	0.03 ± 0.01	20.1 ± 4.8	BHK-21	—	—	—	21
Arbidol	E2	G82R	LR2006 OPY1 (ECSA)	12.2 ± 2.2	376	MRC5	—	—	—	22
Suramin (R)	E2	N5R, H18Q	CHIKV-LS3	79 ± 11.6	>1,000	VeroE6	0611aTw, 0810bTw, 0706aTw (Asian)	Reduced viral load, foot swelling, and histopathologic lesions	C57BL/6	25, 28, 29
Picolinic acid	C	—	DRDE-07 (ECSA)	60% inhibition with 2 mM dose	n.s.	Vero	—	—	—	30
Amantadine	6k	—	S27 (ECSA)	29.5	>200	Vero	—	—	—	32
Chloroquine (R)	—	—	DRDE-06 (ECSA)	7.0 ± 1.5	>260	Vero	—	—	—	38
Doxycycline (R)	—	—	n.s. (ECSA)	10.95 ± 2.12	>100	Vero	061573 (ECSA)	No significant reduction in viral titer or pathology	ICR	39
Curcumin	—	—	LR06-049 (ECSA)	3.89	11.6	HeLa	—	—	—	41
Niclosamide	—	—	S27 (ECSA)	0.95 ± 0.22	>20	BHK-21	—	—	—	42
Nitazoxanide	—	—	S27 (ECSA)	2.96 ± 0.18	>25	BHK-21	—	—	—	42
Apigenin	—	—	LR2006 OPY1 (ECSA)	70.8	>200	BHK-21	—	—	—	43
FL3	—	—	Clinical isolate (ECSA)	0.0224	0.119	HEK-293T	—	—	—	44

a CC_50_, 50% cytotoxic concentration; EC_50_, 50% effective concentration; n.s., not specified; —, not determined/not done (*in vivo* studies); R, repurposed compound. The numbering of mutations that provide resistance is based on the CHIKV genome sequence of the strain indicated in the table, unless indicated otherwise.

b If the study described a family/class of compounds with antiviral activity, only the antiviral activity of the most potent and/or the most representative compound is reported.

c Only compounds for which the antiviral activity was tested using infectious CHIKV are included; compounds identified using only replicon/surrogate systems for which confirmatory experiments with infectious CHIKV were lacking are excluded.

d When a compound showed activity in multiple cell lines and against multiple CHIKV strains, the value corresponding to the best activity (with corresponding cell line) is reported.

**TABLE 2:** Compounds targeting CHIKV replication^*a*^

Compound^*b*^	Viral target	Resistance mutation(s)	*In vitro* efficacy				*In vivo* efficacy			Reference(s)
			CHIKV strain (genotype)^*c*^	EC_50_ (μM) or other readout^*d*^	CC_50_ (μM)	Cell line	CHIKV strain (genotype)	Efficacy	Mouse model	
MADTP-314 (N) (DA)	nsP1	P34S	IO 899 (ECSA)	26 ± 11	>743	Vero	—	—	—	53–55
CHVB-032 (N) (DA)	nsP1	S454G, W456R	IO 899 (ECSA)	2.7	>75	Vero	—	—	—	56, 57
6′-β-Fluoro-homoaristeromycin (N, NA) (DA)	nsP1	G230R, K299E	CHIKV-LS3	0.12 ± 0.04	>250	VeroE6	—	—	—	58, 59
6′-Fluoro-homoneplanocin A (N, NA) (DA)	nsP1	G230R, K299E	CHIKV-LS3	0.18 ± 0.11	>250	VeroE6	—	—	—	59
Difluoromethylornithine (R) (HT)	nsP1	G230R, V326M (nsP1) + *524R (nsP3)	LR06-049 (ECSA)	3 log_10_ reduction in titer with 500 μM dose	>500	BHK-21	LR06-049 (ECSA)	Modest reduction in CHIKV titers	C57BL/6	60, 61, 121
Mycophenolic acid (R) (HT)	nsP1	S23N, V302M (SINV)	DRDE-06 (ECSA)	0.21 ± 0.06	30 ± 3.1	Vero	—	—	—	62, 63, 65
Ribavirin (NA) (HT/DA)	nsP1/nsP4	(Q21K), S23N, V302M (SINV)/C483Y (CHIKV)	Ross C347 (ECSA)	341.1	>30,000	Vero	—	—	—	63, 99, 104
Sofosbuvir (NA) (DA)	nsP4	—	n.s. (Asian)	2.7 ± 0.5	402 ± 32	Huh-7	n.s.	Reduced CHIKV-induced edema and viral replication	Swiss Webster mouse arthralgia model	94
β-d-*N*^4^-hydroxycytidine (NA) (DA)	nsP4	P187S, A189V, I190T (VEEV)	LR2006 OPY1 (ECSA)	0.2 ± 0.1	7.7	Vero	—	—	—	95, 96
Favipiravir (NA) (DA)	nsP4	K291R	IO 899 (ECSA)	25 ± 3	>636	Vero-A	S27 (ECSA)	Reduced mortality by >50% and improved disease outcome	AG129 lethal model	97
							IO 899 (ECSA)	Reduced viral replication in joints and extremities during acute phase	C57BL/6J	98
Digoxin (R) (HT)	nsP4	V209I	SL15649 (ECSA)	0.048	>10	U2OS	—	—	—	100
HS-10 (HT)	nsP4	—	Ross (ECSA)	>2 log_10_ reduction in titer with 6.25 μM dose	>100	HEK-293T	DMERI09/08 (ECSA)	Reduced inflammation and viremia	SvA129	101
SNX-2112 (HT)	nsP4	—	Ross (ECSA)	>2 log_10_ reduction in titer with 6.25 μM dose	>100	HEK-293T	DMERI09/08 (ECSA)	Reduced inflammation and viremia	SvA129	101
6-Azauridine (R) (NA) (HT/DA)	—	—	Ross C347 (ECSA)	0.8	208	Vero	—	—	—	104
RYL-634	—	—	n.s.	0.26	>2.5	Vero	—	—	—	105
Atovaquone (R)	—	—	LR06-049 (ECSA)	<0.75	>11.25	Vero	—	—	—	106
Berberine	—	—	LR2006 OPY1 (ECSA)	>5 log_10_ reduction in titer with 3 μM dose	>100	BHK-21	LR2006 OPY1 (ECSA)	Reduced inflammation and joint swelling	C57BL/6J	107, 109
Ivermectin (R)	—	—	LR2006 OPY1 (ECSA)	>4 log_10_ reduction in titer with 3 μM dose	37.9 ± 7.6	BHK-21	—	—	—	107
Abamectin (R)	—	—	LR2006 OPY1 (ECSA)	>4 log_10_ reduction in titer with 3 μM dose	28.2 ± 1.1	BHK-21	—	—	—	107
Harringtonine	—	—	0708 (ECSA)	0.24	>100	BHK-21	—	—	—	110
Silymarin	—	—	MY/065/08/FN295485 (ECSA)	35	881	Vero	—	—	—	111
Andrographolide	—	—	0708 (ECSA)	77.39	1,098	HepG2	—	—	—	115
Micafungin (R)	—	—	S27 (ECSA)	20.6 ± 1.7	>100	U2OS	—	—	—	116
MBZM-N-IBT	—	—	S27 (ECSA)	38.7	>800	Vero	—	—	—	117
Imipramine (R)	—	—	LR2006 OPY1 (ECSA)	3 log_10_ reduction in titer with 75 μM dose	>100	HFF1	—	—	—	112
Tomatidine	—	—	LR2006 OPY1 (ECSA)	1.3	156	Huh-7	—	—	—	113
Silvestrol (HT)	—	—	IO 899 (ECSA)	0.00189	>0.03	HEK-293T	—	—	—	114

a n.s., not specified; —, not determined/not done (*in vivo* studies); N, novel; NA, nucleoside analogue; R, repurposed compound; VEEV, Venezuelan equine encephalitis virus. The numbering of mutations that provide resistance is based on the CHIKV genome sequence of the strain indicated in the table, unless indicated otherwise. DA, direct-acting compounds; HT, host-targeting compounds; HT/DA, both host-targeting and direct-acting compounds.

b If the study described a family/class of compounds with antiviral activity, only the antiviral activity of the most potent and/or the most representative compound is reported.

c Only compounds for which the antiviral activity was tested using infectious CHIKV are included; compounds identified using only replicon/surrogate systems for which confirmatory experiments with infectious CHIKV were lacking were excluded.

d Where a compound showed activity in multiple cell lines and against multiple CHIKV strains, the best value (with corresponding cell line) is reported.

**TABLE 3:** CHIKV inhibitors identified by *in silico* approaches (molecular docking, homology modeling, pharmacophore modeling)

Compound	Viral target	Resistance mutation(s)	Confirmed in enzymatic assays?	Confirmed in infected cells?	*In vitro* efficacy				*In vivo* efficacy			Reference
					CHIKV strain (genotype)	EC_50_^*b*^	CC_50_ (μM)	Cell line	CHIKV strain (genotype)	Efficacy	Mouse model	
25	nsP2	—^*a*^	No	Yes	IO 899 (ECSA)	3.2 ± 1.8	101 ± 50	Vero	—	—	—	78
7	nsP2	—	No	Yes	LR2006 OPY1 (ECSA)	0.42	>100	Vero	—	—	—	79
8	nsP2	—	Yes	Yes	LR2006 OPY1 (ECSA)	∼1.5	>200	BHK-21	—	—	—	80
3a	nsP2	—	No	Yes	n.s.	8.76 μg/ml	n.s.	Vero	—	—	—	81
4b	nsP2	—	No	Yes	n.s.	8.94 μg/ml	n.s.	Vero	—	—	—	81
Baicalin	nsP3	—	No	Yes	MY/065/08/FN295485 (ECSA)	5	>600	Vero	—	—	—	90

a —, not determined/not done (*in vivo* studies).

b Data represent micromoles unless otherwise indicated.

**TABLE 4:** CHIKV inhibitors identified in *in vitro* biochemical assay/assays with purified protein^*a*^

Compound	Viral target	Resistance mutation(s)	Confirmed in infected cells?	*In vitro* efficacy				*In vivo* efficacy			Reference or source
				CHIKV strain (genotype)	EC_50_ (μM)	CC_50_ (μM)	Cell line	CHIKV strain (genotype)	Efficacy	Mouse model	
5-Iodotubercidin (NA)	nsP1	—	Yes	Clinical isolate 119067	0.409	>50	Vero	—	—	—	66
lobaric acid	nsP1	—	Yes	LR2006 OPY1 (ECSA)	9.9 ± 2.6	76.3 ± 2.1	BHK	—	—	—	67
Sinefungin	nsP1	—	Yes	CHIKV LS3	184.9 ± 38.4	>1,000	VeroE6	—	—	—	66, 122; unpublished data^*b*^

a For compounds identified using *in silico* approaches, only those compounds for which the antiviral activity was demonstrated in cell culture using infectious CHIKV are reported; all other compounds for which activity was claimed against CHIKV from *in silico* screens, but for which no activity in cell-based assays was reported, are excluded from this table. —, not determined/not done (*in vivo* studies).

b K. Kovacikova, M. G. Gonzalez, J. Reguera, Eric J. Snijder, Gerard J. P. van Westen, and Martijn J. van Hemert, unpublished data.

## STRATEGIES FOR ANTI-CHIKV DRUG DISCOVERY AND DESIGN

Considering the global distribution of CHIKV and its mosquito vectors, the potential for additional spread, and the impact on human health, development of preventive measures is imperative. Several approaches have been used for identification of potential CHIKV inhibitors, including cell-based high-throughput screening (HTS) campaigns, rational and structure-based drug design using crystal structures, and homology modeling of viral proteins. One of the conventional approaches for CHIKV drug discovery uses cell-based screening with readouts that measure the virus-induced CPE. The CPE reduction assay screens can provide information on antiviral activity of compounds, and their cytotoxicity can be assessed in parallel using uninfected cells in the same plate. Such screens are often deployed to test known clinically approved drugs, in a process referred to as drug repurposing. The advantages of this route are decreased costs related to drug approval and an accelerated process leading to potential licensing of the compound. Due to increased computational power, HTS has emerged recently as an efficient process for screening thousands of compounds from large compound libraries, including FDA-approved and novel molecules. The emergence of computer-aided drug design has also greatly contributed to the development of CHIKV inhibitors. These approaches are based on the structure of a viral protein to perform *in silico* virtual screens. Compounds identified by *in silico* computer-based screening can be further optimized by acquiring an understanding of the compound’s structure-activity relationship (SAR), and improved derivatives can be synthesized for validation in enzymatic and cell-based assays. However, the utility of computer-aided design for compounds targeting the CHIKV replicase is limited because, thus far, only the structures of the N-terminal region and C-terminal region of nonstructural protein 2 (nsP2), representing the RNA helicase and protease domains, respectively, and the N-terminal macrodomain of nsP3 have been resolved (11–13). More opportunities for molecular docking studies arise from analyses of CHIKV structural proteins, as the structures for the envelope (E) proteins and the capsid (C) protein have been determined. Although the crystal structures for complete CHIKV nsPs are not yet available, researchers have used various purification methods to obtain enzymatically active recombinant nsPs for use in cell-free assays. Validation of compounds originating from *in silico* virtual screens in enzymatic assays performed with purified proteins is especially important for confirmation of target specificity. In cell-based assays, resistance selection in the presence of (suboptimal concentrations of) a compound has been widely used to identify the viral target of such compounds. Tables 1 and 2 provide a comprehensive overview of all resistance mutations that have been identified so far, information which—together with cross-resistance studies—can aid in the elucidation of the mode of action of compounds that might be identified in future screening efforts. Besides the compound’s mode of action, an understanding of molecular determinants of resistance can provide useful information about the virus replication cycle and pathogenesis.

## COMPOUNDS TARGETING CHIKV ENTRY AND EGRESS

The alphavirus virion is composed of a nucleocapsid core with T=4 icosahedral symmetry surrounded by a host-derived lipid bilayer which is decorated with 80 trimeric spikes of E1-E2 heterodimers (14–16). The E2 protein mediates viral entry by attachment to the receptors on the cell surface (17). This process is followed by clathrin-mediated endocytosis, which delivers the viral particle to the early endosomes (18). The low pH in the endosomal compartment triggers conformational changes in the E1-E2 heterodimer and results in the insertion of the E1 fusion protein into the endosomal membrane. More specifically, the E1 glycoprotein is converted from a nonfusogenic form to a highly stable fusogenic E1 homotrimer (19, 20). This event ultimately creates a fusion pore and releases the nucleocapsid into the cytosol. Compounds that exhibit antiviral activity against entry, fusion, and egress of CHIKV are listed in Table 1.

## ENVELOPE PROTEIN E1

Obatoclax, an anticancer compound, was found to inhibit CHIKV infection early in the replication cycle by neutralizing the acidic endosomal environment required for fusion. The L369I mutation in domain III of the E1 fusion glycoprotein conferred at least partial resistance to obatoclax and generated resistant viruses with enhanced fusogenic potential (21).

## ENVELOPE PROTEIN E2

Arbidol/umifenovir has been licensed as an anti-influenza agent in both Russia and China and inhibits a wide range of viruses. Arbidol inhibits early stages of the CHIKV replication cycle, which was confirmed by selection of an arbidol-resistant variant carrying a G407R mutation in the structural polyprotein (22), which corresponds to a G82R mutation in the E2 glycoprotein. This mutation also caused attenuation of CHIKV vaccine strain 181/25, presumably by increasing interactions with glycosaminoglycans on the host cell surface (23). Arbidol derivatives with increased potency and selective inhibition of CHIKV have been developed, but the precise mechanism of action of these compounds remains unresolved (24).

Suramin is an approved drug for treatment of parasitic infections in humans and was shown to inhibit CHIKV entry in three independent studies (25–27). The compound likely influences CHIKV attachment to cells and may prevent conformational changes of the E1/E2 heterodimer that are required for viral fusion. Selection of suramin-resistant variants revealed that the mutations N5R and H18Q in the E2 glycoprotein cause some resistance to the compound. Molecular docking with the mature CHIKV spike suggested that suramin interacts with the N-terminal loop and domain A in the E2 glycoprotein, an interaction that would negatively affect virion binding to the receptor (28). Suramin treatment of C57BL/6 mice infected with different clinical isolates of CHIKV ameliorated CHIKV-induced foot swelling, inflammation, and cartilage damage (29).

## CAPSID PROTEIN

Picolinic acid was found to bind to the hydrophobic pocket of the C protein, which might inhibit the C protein’s interaction with the cytoplasmic domain of the E2 glycoprotein. The antiviral activity of picolinic acid was also confirmed in CHIKV-infected cells, although the inhibitory concentration was rather high for clinical applications (30).

## 6K

The 6K protein belongs to the family of viroporins or ion channel-forming proteins. It is a highly hydrophobic protein with membrane fusogenic properties. Viroporins enable movement of small molecules and ions across membranes, which can be important during viral entry, replication, and egress. The potential of 6K to serve as a therapeutic target is illustrated by the drug amantadine, a well-known influenza inhibitor that targets the ion channel-forming M2 protein of influenza viruses (31). Amantadine inhibited ion channel activity and altered particle morphology in biophysical systems. Mechanistically, 6K likely needs to interact with E2 for its delivery to the plasma membrane where it forms an ion channel. The antiviral effect of amantadine on CHIKV was also confirmed in infected cells (32).

## DRUGS TARGETING CHIKV ENTRY WITH AN UNCHARACTERIZED MODE OF ACTION

Chloroquine (CHL) is an old antimalaria drug with a broad range of antiviral activities against a variety of viruses. CHL had been deployed in clinical trials long before its anti-CHIKV activity was established in cell culture. The rationale for this unusual strategy was that CHL had conferred benefits in lessening joint inflammation in patients with rheumatoid arthritis during trials in the late 1950s (33). The efficacy of CHL phosphate was first investigated in a small patient cohort, which led to an alleviation of patient symptoms and justified its further use for the treatment of CHIKV-associated arthritis (34). However, the putative benefits of CHL for the treatment of acute CHIKV infection were disproved in the “CuraChik” trial conducted during the 2005–2006 La Reunion epidemic (https://clinicaltrials.gov/ct2/show/NCT00391313). Moreover, CHL-treated patients had more frequent complaints of arthralgia than placebo recipients (35). In an Indian trial during the 2006 CHIKV epidemic, CHL also did not yield benefits in relieving symptoms of musculoskeletal pain and arthritis compared to the commonly used nonsteroidal anti-inflammatory drug meloxicam (36). These and other studies supported the hypothesis that CHL might enhance viral replication, which was later demonstrated *in vivo* in CHL-treated BALB/c mice infected with another arthritogenic alphavirus, Semliki Forest virus (37). In CHIKV-infected cells, CHL seems to block or delay virus internalization depending on the time of treatment. It is effective at early stages of viral infection, likely by impairing cell-virus surface interactions and blocking endosomal acidification (38).

Doxycycline is a tetracycline antibiotic used for treatment of bacterial infections that has also shown promising anti-CHIKV activity. It was postulated that the anti-CHIKV activity of doxycycline is directed toward viral entry rather than viral replication. Docking studies with the CHIKV nsP2 cysteine protease and E2 glycoprotein indicated that the compound could bind to both these viral targets. However, cell-based assays confirmed that doxycycline inhibits viral entry, likely by impairing conformational changes in the E2 glycoprotein. Treatment of adult ICR mice with doxycycline alone did not result in an improved outcome in comparison with combination treatment with ribavirin (39).

Curcumin is a turmeric plant extract that has been used for treatment of gastrointestinal disorders in Asia. The antiviral effect of curcumin on CHIKV has been demonstrated using pseudotyped viral particles (40), and a study using wild-type CHIKV subsequently showed that curcumin reduces the infectivity of CHIKV particles and their binding at the cell surface (41). A CHIKV insect cell fusion inhibition assay was used to screen for fusion inhibitors and identified two compounds, niclosamide and nitazoxanide, as prospective CHIKV inhibitors. In other assays, both compounds were confirmed to inhibit CHIKV entry and suppress cell-to-cell transmission (42).

Apigenin, a natural compound with a 5,7-dihydroxyflavone structure, has shown moderate anti-CHIKV activity. Flavonoids have been previously reported to suppress the entry pathway of members of other virus families. However, the flavonoids tested against CHIKV, among which apigenin was the most potent, strongly inhibited CHIKV replicon levels and had no effect in an Semliki Forest virus (SFV) entry assay (43), suggesting that they do not target entry. Synthetic flavaglines such as FL3 were based on a class of naturally occurring plant compounds with activity in the low nanomolar range. FL3 inhibited CHIKV infection at the entry step by serving as a prohibitin ligand and disrupting the interaction between CHIKV and the prohibitin receptor (44).

## COMPOUNDS TARGETING CHIKV REPLICATION

The incoming alphavirus genomic RNA is translated into two polyproteins: P123 and P1234. P123 is the more abundant of the two, and P1234 arises as a result of the translational read-through of an opal stop codon (with about 10% efficiency) at the end of nsP3 coding sequence (45). These polyproteins are processed by the protease domain of nsp2. Cleavage intermediates as well as fully cleaved individual viral nsPs play specific roles in CHIKV (−) and (+) strand RNA synthesis. The functions of nsPs have been largely characterized by using recombinant viruses with mutations in nsPs in biochemical assays and by *in silico* identification of enzymatic sequence motifs. CHIKV inhibitors targeting individual nsPs are described in Tables 2, 3, and 4.

## NONSTRUCTURAL PROTEIN 1 (NSP1)

Alphavirus nsP1 (535 amino acids [aa]) is a viral mRNA capping enzyme with guanine-N7-methyltransferase (MTase) and guanylyltransferase (GTase) activities responsible for capping of the 5′ ends of the newly synthesized genomic 42S mRNA and 26S subgenomic mRNA. The MTase catalyzes the transfer of the methyl group from S-adenosylmethionine (SAM) to the N7 position of a GTP molecule, forming m^7^GTP and releasing S-adenosylhomocysteine (SAH) as a by-product (46). The GTase binds the m^7^GTP and forms a covalent intermediate, m^7^GMP-nsP1, while releasing a pyrophosphate (PPi) (47). The m^7^GMP is then transferred to the 5′-diphosphate RNA, which is generated through the RNA 5′ triphosphatase activity of nsP2 (48), to create a methylated cap structure at the 5′ terminus. This cap structure is essential for viral mRNA translation and prevents mRNA degradation by host 5′-exonucleases. The middle part of nsP1, spanning amino acid residues 245 to 264, contains an amphipathic helix responsible for association of the alphavirus replication complex (RC) with membranes (49). Specifically, the presence of amphipathic helix and palmitoylated cysteines 417 to 419 (50) allows nsP1 and nsP1-containing replication complexes to anchor to cholesterol-enriched membrane microdomains (51). Importantly, site-directed mutagenesis of conserved residues in alphavirus nsP1 indicated that abrogation of nsP1 enzymatic activities is detrimental for virus replication (52).

In recent years, the number of reports describing an inhibitory effect of molecules specifically targeting nsP1 functions has substantially increased (Tables 2 and 4). Owing to its uniquely viral enzymatic activities, nsP1 represents an excellent target for antiviral compounds, while not affecting host cell mRNA capping, which proceeds through a fundamentally different mechanism. The 3-aryl-[1,2,3]triazolo[4,5-d]pyrimidin-7(6×H)-ones were first reported as a class of potent and selective inhibitors of CHIKV replication that target nsP1, with MADTP-314 as the prototype compound (53, 54). A resistance selection procedure performed with MADTP-314 and subsequent reverse genetics indicated that the single-amino-acid substitution P34S in the N-terminal part of nsP1 was responsible for resistance to MADTP-314 and several analogues of this compound (55). Recently, the 2-(4-[phenylsulfonyl]piperazine-1-yl)pyrimidine analogues known as the CHVB series, with CHVB-032 as the prototypical compound, were identified as potent and selective CHIKV inhibitors (56). Reverse genetics revealed two mutations in the C-terminal region of nsP1, namely, S454G and W456R, to be responsible for resistance to CHVB-032 and its analogues. Interestingly, the two families of compounds seem to target the same nsP1 functions, as the MADTP-resistant nsP1-P34S mutant is cross-resistant to CHVB-032 and its analogue CHVB-066 and, vice versa, the CHVB-resistant nsp1-S454G+W456R mutant is cross-resistant to a MADTP-314 analogue, MADTP-372 (57). Yet another phenotypic compound screen identified 6′-β-fluoro-homoaristeromycin (FHA) and 6′-fluoro-homoneplanocin A (FHNA) as potent CHIKV inhibitors with a very high therapeutic index (58, 59). The mutations G230R and K299E in nsP1 were identified by resistance selection and reverse genetics to confer resistance to both FHA and FHNA (59). Difluoromethylornithine (DFMO), an inhibitor of ornithine decarboxylase 1, was shown to have a broad-spectrum antiviral effect on a variety of RNA viruses, including CHIKV (60). Interestingly, CHIKV can overcome polyamine depletion by acquiring mutations in nsP1. The combination of the G230R and V326M mutations in CHIKV nsP1 and *524R in nsP3 was essential to confer resistance to DFMO (61). Despite its promising *in vitro* effects, there was little protection against CHIKV-induced disease in C57BL/6 mice that were fed DFMO in their drinking water prior to infection (60). Mycophenolic acid (MPA) is a well-known immunosuppressive agent, and ribavirin is a broad-spectrum guanosine analogue with immunomodulatory properties. Both compounds target the host enzyme IMP dehydrogenase (IMPDH), which is important for the *de novo* synthesis of GMP and the regulation of intracellular GTP levels. GTP is critical for at least two processes in alphavirus replication: it serves as a methyl acceptor molecule during mRNA capping and as a building block in nsP4-mediated RNA synthesis, as described below. The anti-CHIKV activity of MPA was shown to be associated with the depletion of the guanosine pool in cell culture (62). Similarly to FHNA, treatment with MPA resulted in a release of virions with reduced specific infectivity (PFU per genome copy number). Earlier studies with Sindbis virus (SINV) mapped the mutations responsible for the MPA-resistant phenotype to the region encoding nsP1. Viruses with the MPA-resistant phenotype were also resistant to ribavirin (63, 64). Later on, in-depth reverse genetics studies with a SINV cDNA clone demonstrated that only the mutations S23N and V302M in nsP1 were essential for MPA resistance (65). To date, CHIKV nsP1 crystal structure has not been available, which makes it hard to appreciate the structural context of the various compound-resistant mutations and understand the molecular mechanisms underlying drug resistance. Taking the data together, the majority of CHIKV nsP1 inhibitors for which the molecular determinants of resistance were studied require a combination of two nsP1 mutations for compound-specific resistance. Since singular mutations did not cause resistance in most cases, the chance of the emergence of drug-resistant CHIKV variants during treatment appears to be low for these compounds.

Other CHIKV nsP1 inhibitors have been identified by using purified CHIKV nsP1 in enzymatic assays (Table 4). 5-Iodotubercidin, an adenosine analogue, was recently discovered by screening with a novel capillary electrophoresis-based assay for MTase activity of CHIKV nsP1. The activity of the compound in this enzymatic assay was validated in cell culture (66). This enzymatic assay uncouples the MTase and GTase activities and thus can be used to identify specific alphavirus nsP1 MTase inhibitors. A fluorescence polarization-based assay measuring competition for the GTP-binding site was used in a large HTS screen that identified lobaric acid as a potent CHIKV nsP1 inhibitor, which was also validated using live virus in cell-based assays (67). Since GTP binding is essential to perform the MTase step in mRNA capping, the assay identifies competitive MTase inhibitors.

## NONSTRUCTURAL PROTEIN 2 (NSP2)

Alphavirus nsP2 (798 aa) is a multifunctional protein which possesses several enzymatic activities, including nucleoside triphosphatase (NTPase) (68, 69), helicase (70), and RNA 5′ triphosphatase (RTPase) activity (48) in the N-terminal part of the protein and protease activity (71, 72) and a SAM-dependent RNA methyltransferase-like (SAM MTase-like) domain in the C-terminal part of nsP2. The NTPase and helicase functions are important for unwinding double-stranded RNA during CHIKV replication, and the RTPase activity removes the γ-phosphate from the 5′ end of the RNA before the transfer of the cap-0 structure. The nsP2 protease activity is responsible for nsp123 and nsp1234 polyprotein processing (72). In the case of Old World alphaviruses, nsP2 can also induce host transcriptional shutoff and cytopathic effects (73). During host shutoff, nsP2 translocates to the nuclei of vertebrate cells to induce polyubiquitination of the catalytic subunit of the DNA-dependent RNA polymerase II, RPB1, and in this way subverts the cellular antiviral response (74, 75). The C-terminal SAM MTase-like domain plays a critical role in the nuclear function of alphavirus nsP2 (76) and inhibits the interferon response (77).

The crystal structure of the nsP2 protease domain has been solved, and the protein is now used as an important target for antiviral development using computer-aided drug design (Table 3). A virtual screening campaign analyzing a library of commercially available compounds using a homology model of CHIKV nsP2 protease identified compound 1 as an initial hit. The compound displayed anti-CHIKV activity in cell-based assays, and SAR studies on 25 structural analogues yielded compound 25, which showed improved efficacy compared to and lower cytotoxicity than lead compound 1 (78). Another study reported on five arylalkylidene derivates of 1,3-thiazolidin-4-one with anti-CHIKV activity in the low micromolar range, with compound 7 being the most potent. Using molecular docking, the compounds were shown to partially interact with the crystal structure of the nsP2 protease (79). Although the above-mentioned studies identified novel nsP2-targeting molecules, they did not provide experimental evidence, e.g., obtained using enzymatic assays, to demonstrate that nsP2 was *de facto* the target of these compounds. Computer-aided drug design was combined with cell-free assays for target validation of a set of 12 compounds designed against the CHIKV nsP2 protease using target-based modeling. The most promising compound, compound 8, potently inhibited CHIKV replication in cell culture and was moderately active in the protease assay with recombinant CHIKV nsP2 (80). This illustrates the importance of confirming *in silico* predictions in enzymatic assays with purified protein as virtual binding does not always correlate with inhibition of enzymatic activity *in vitro*. Other inhibitors targeting nsP2 include small peptidomimetics discovered using a unique approach of quantum mechanics-based ligand descriptors. Compounds with lower molecular weight displayed greater inhibitory activity, likely due to superior access to the target pocket (81).

## NONSTRUCTURAL PROTEIN 3 (NSP3)

The functional role of alphavirus nsP3 (530 aa) is the least defined among all CHIKV nsPs. Three domains can be distinguished in nsP3: an N-terminal macrodomain (13), a Zn-binding alphavirus unique domain (AUD) (82), and a C-terminal hypervariable domain (HVD) (83). While the N-terminal part of nsP3 is well conserved, the C-terminal HVD has very low sequence similarity even between closely related alphaviruses. Alphaviruses use their HVDs to recruit the RNA-binding proteins typically found in stress granules, such as the G3BP proteins used by CHIKV, for the formation of pre-RCs that promote viral replication (84). Other cellular proteins from different families, specific for virus species and cell types, have been found to interact with nsP3 HVD (85). They function as the major determinants of cell specificity during viral replication. The nsP3 macrodomain affects various critical processes in the alphavirus replication cycle, including nsP3 phosphorylation, (−) strand RNA synthesis, host translational shutoff, and virulence (86). Importantly, ADP ribosylation of cellular proteins, a posttranslational modification involved in a variety of cellular processes, is regulated by the nsP3 macrodomain. The nsP3 macrodomain possesses both ADP-ribosyl-binding and ADP-ribosylhydrolase activities, by which it binds ADP-ribose and hydrolyzes ADP-ribosylated residues on cellular proteins. The nsP3 macrodomain-mediated ADP-ribosyl binding is necessary for initiating nsP synthesis and establishing RCs, while the ADP-ribosylhydrolase activity is important for amplification of RCs. Thus, interaction of nsP3 macrodomain with ADP-ribosylated proteins is required for efficient alphavirus replication (86). Besides that, the ADP-ribosylhydrolase activity is a determinant of neurovirulence in mice (87). The nsP3 AUD appears to be a multifunctional domain that plays a role in virus genome replication (88).

To date, only a few small-molecule inhibitors targeting CHIKV nsP3 have been reported, perhaps due to the enigmatic role of nsP3 in viral replication. However, in-depth characterization of the nsP3 functional domains in recent years has contributed to the exploitation of nsP3 as a potential drug target. Given its highly conserved nature and the available crystal structure, the macrodomain represents an ideal site for development of specific anti-CHIKV antivirals. Baicalin is one of the very few compounds that have been shown to interact with nsP3 using a computational approach (89) with a confirmed anti-CHIKV activity in cell culture (90) (Table 3). However, the latter study revealed that baicalin inhibited early stages of CHIKV replication and has strong virucidal activity. Moreover, baicalin was shown to interact with the CHIKV E glycoprotein using molecular docking, which led to discrepancy with the previous study suggesting that nsP3 is the viral target of this compound. This again illustrates that it should become the norm that the antiviral activity and mode of action of small-molecule inhibitors discovered using computer-aided drug design are validated in cell-based assays.

## NONSTRUCTURAL PROTEIN 4 (NSP4)

The nsP4 protein (611 aa) is the most conserved protein in the alphavirus family that functions as an RNA-dependent RNA polymerase (RdRp) responsible for replication of 49S genomic (+) strand RNA and transcription of the 26S single guide RNA (sgRNA). In the early phase of the replication cycle, nsP4, together with P123, is part of an early replication complex (RC) that is responsible for the synthesis of full-length (−) strand RNA that serves as the template for synthesis of genomic and sgRNA. Following polyprotein processing, the late RC consisting of fully cleaved nsP1 to nsP4 mediates the synthesis of genomic RNA and 26S sgRNA (91). NsP4 also possesses a terminal adenylyltransferase (TATase) activity that catalyzes the addition of a poly(A) tail to the 3′ end of the genome (92). In addition, nsP4 contains the signature GDD motif of the catalytic core of RdRp and mutation of both aspartate residues to alanine results in a complete loss of TATase activity (92).

Many RdRp inhibitors are nucleoside analogues. Because RdRp activity is absent from host cells, it represents a suitable target for development of antiviral agents (Table 2). Sofosbuvir is a uridine analogue that is clinically approved for the treatment of hepatitis C virus infection. It is administered as a UMP prodrug that needs to be metabolized to yield the pharmacologically active compound sofosbuvir triphosphate (93). nsP4 is the predicted target of sofosbuvir in CHIKV-infected cells, based on molecular docking of the compound on the putative CHIKV nsP4 model. Treatment of hepatoma cells with sofosbuvir decreased CHIKV replication *in vitro*, and its administration to infected adult Swiss mice resulted in reduced arthralgia-related paw inflammation (94).

β-d-*N*^4^-Hydroxycytidine (NHC) is a nucleoside analogue that inhibits CHIKV replication after it is converted to its active form, NHC triphosphate. However, a direct relationship between NHC and its mode of action in CHIKV-infected cells has not yet been established. It was proposed that NHC may interfere with CHIKV replication through chain termination or mutagenesis (95). Studies with Venezuelan equine encephalitis virus, a New World alphavirus, demonstrated that resistance to NHC develops very inefficiently and is determined by a synergistic effect of multiple mutations in nsP4. In particular, three nsP4-specific mutations, namely, P187S, A189V, and I190T, located in the index finger domain of the predicted nsP4 structure, are thought to be responsible for resistance to NHC. Interestingly, the NHC-resistant phenotype can revert to the wild type after incorporation of the A201V mutation in nsP4, which has a negative effect on viral resistance to NHC (96).

Initially developed as an anti-influenza virus inhibitor, favipiravir (T-705) is a broad-spectrum nucleoside analogue that has also shown inhibitory activity against CHIKV (97). All of the favipiravir-resistant variants acquired the unique K291R mutation in nsP4, which is a highly conserved residue in the F1 motif of the RdRps of (+) strand RNA viruses (97). In addition, treatment with favipiravir reduced viral loads in the brain of infected AG129 mice and protected them from severe neurological disease. Favipiravir was also tested in immunocompetent C57BL/6J mice during the acute and chronic phases of CHIKV infection. Treatment with favipiravir during the acute phase rendered viral RNA, viral antigens, and infectious particles undetectable. However, such a reduction was not observed upon favipiravir treatment during the chronic phase (98). Given that full-length CHIKV RNA could not be recovered from chronically infected mice, the data suggest that the viral RNA can be defective and unable to form infectious particles, corroborating earlier findings from studies performed with patient material.

As discussed above, ribavirin can block CHIKV replication by at least two different mechanisms, one of which operates through RdRp inhibition. CHIKV passaging in the presence of ribavirin yielded a high-fidelity mutant containing a C483Y mutation in nsP4 (99). Interestingly, this CHIKV mutant generated populations with restricted genetic diversity, which appears to have resulted from a novel mechanism that differs from those typical for nucleoside analogues, such as chain termination or lethal mutagenesis. The analysis of the effect of resistance mutations on the three-dimensional (3-D) structure of nsP4 was based on homology modeling, since a crystal structure of the CHIKV RdRp is not yet available.

Despite the majority of nsP4-targeting compounds belonging to well-known classes of nucleoside analogues, compound screens have identified other potential drug candidates. For example, an HTS of advanced clinical candidates identified digoxin, a cardiac glycoside that antagonizes the sodium-potassium ATPase, as a potent CHIKV inhibitor. The V209I mutation in nsP4, situated in a well-conserved region of nsP4, was found to play a pivotal role in the development of digoxin-mediated resistance (100). Cytoplasmic proteins involved in CHIKV replication could also be targeted due to their direct interaction with CHIKV proteins. Hsp90 proteins are host proteins that serve as molecular chaperones with a wide array of functions. The cytoplasmic subunit HsP90α was shown to be the predominant interacting partner of nsP4 in coimmunoprecipitation experiments. Hsp90 proteins seem to play a role in stabilizing RCs during alphaviral infections. Hsp90 inhibitors HS-10 and SNX-2112 inhibited CHIKV replication in cell culture, and they reduced CHIKV-induced joint swelling and viral load in infected mice (101). Direct evidence leading to nsP4 being identified as the viral target of these compounds is, however, still lacking.

## INHIBITORS OF CHIKV REPLICATION WITH AN UNDEFINED TARGET

6-Azauridine is a broad-spectrum nucleoside analogue that has been widely used in patients for other indications. Its metabolite 6-azauridine 5′monophosphate was previously reported to inhibit the replication of several DNA and RNA viruses via targeting of the host orotidylic acid decarboxylase (102). Others have proposed a different mechanism of action, based on interference with cellular UTP metabolism, leading to the so-called “error” catastrophe (103). Multiple studies have demonstrated that 6-azauridine potently inhibits CHIKV replication, but its viral target has not been determined (104).

Analysis of a chemical library has identified RYL-634, a novel potent broad-spectrum small-molecule inhibitor with antiviral activity against many pathogenic viruses, including CHIKV. Dihydroorotate dehydrogenase (DHODH) was validated as the target enzyme of RYL-634 using activity-based protein profiling (105).

Atovaquone is a ubiquinone analogue and a well-known antimalarial and antiparasitic drug. Previous studies indicated that it can function through inhibition of mitochondrial function or DHODH, the latter being required for the *de novo* synthesis of pyrimidine. Recently, atovaquone was found to inhibit CHIKV replication, but the mechanism underlying its precise mode of action remains unstudied. For Zika virus, it was shown that atovaquone blocks DHODH and thereby leads to the depletion of intracellular nucleotide pools (106).

Another HTS campaign identified berberine, ivermectin, and abamectin as strong inhibitors of CHIKV replication. Ivermectin and abamectin are broad-spectrum antiparasitic drugs used for the treatment of humans and agricultural crops, respectively. Berberine possesses antimicrobial properties, and it has been tested for antiviral activity against a range of viruses, including herpes simplex virus, influenza virus, and cytomegalovirus (107). Further elucidation of its anti-CHIKV effect showed that berberine impairs mitogen-activated protein kinase signaling pathways, although the specific molecular target of berberine remains unknown. Another study found that berberine affects postreplications steps in the CHIKV replication cycle by targeting interactions between genomic RNA and C protein that are required for correct nucleocapsid assembly (108). Moreover, treatment of CHIKV-infected C57BL6/J mice with berberine alleviated the symptoms of CHIKV-induced inflammatory disease (109).

Harringtonine, a cephalotaxine alkaloid, was discovered by screening a natural product compound library and was also found to potently inhibit CHIKV replication. This compound acts on the postentry stage of the CHIKV replication cycle and strongly interferes with CHIKV protein synthesis. It was previously postulated that harringtonine inhibits the host cell translation machinery and thereby leads to the suppression of translation of CHIKV nsPs and structural proteins (110). Silymarin is a flavonoid with anti-CHIKV activity that was also found to exert its antiviral activity at the postentry stage (111). Imipramine is an FDA-approved antidepressant that exerts its antiviral effects at two distinct stages in CHIKV replication, the fusion/entry step and a postfusion replication step. Because optimal fusion reactions and intracellular replication are both dependent on cholesterol, these processes are highly susceptible to imipramine, which is a class II cationic amphiphilic drug targeting the cholesterol trafficking pathway (112). Tomatidine is a natural steroidal alkaloid that interferes with postentry steps in the CHIKV replication cycle (113). Silvestrol is a natural compound that belongs to the flavaglines and is a specific inhibitor of the host RNA helicase eukaryotic initiation factor 4A (eIF4A), which is part of the translation initiation complex. Silvestrol treatment of CHIKV-infected cells delayed the translation of viral proteins and prevented host transcriptional shutoff (114). Andrographolide, a bicyclic diterpenoid lactone (115); micafungin, an antifungal agent (116); and MBZM-N-IBT (117) all inhibit CHIKV replication, but their mechanism of action remains unknown.

## ARE DRUG-RESISTANT MUTANTS ATTENUATED IN MOSQUITOES?

Compared to single-host RNA viruses, the alternating use of insect and mammalian hosts restricts arbovirus adaptation to environmental pressures such as treatment with antiviral compounds. Selection and fitness of new variants are influenced by replication competence in both vertebrate and invertebrate hosts. To date, only a few studies have specifically assessed the fitness of drug-resistant mutants in mosquitoes. Such information would be valuable for selecting an antiviral agent with minimal risk of inducing and spreading drug resistance in the environment. For example, a high-fidelity ribavirin-resistant variant containing the C483Y mutation in nsP4 had lower fitness in Ae. aegypti mosquitoes (99). Likewise, the favipiravir-resistant mutant carrying the K291R mutation in nsP4 also disseminated poorly in the bodies of Ae. aegypti mosquitoes and showed decreased transmission potential, while the MADTP-resistant mutant with the P34S mutation in nsP1 showed the same transmission efficiency as wild-type virus (118). Furthermore, a DFMO-resistant triple mutant carrying the G230R and V326M mutations in nsP1 and the nsP3-opal524R mutation replicated to higher titers in Ae. albopictus mosquitoes than the wild-type virus (61). These studies indicate that drug-resistant mutants can have quite different phenotypes in mosquitoes and emphasize the need to determine their transmission potential for those antiviral drugs intended for clinical use.

## HOW CHOICE OF CELL LINE IN CHIKV ANTIVIRAL DRUG DISCOVERY CAN AFFECT OUTCOME

Vertebrate cells such as BHK-21 (baby hamster kidney) cells or Vero E6 (African green monkey kidney) cells are the most widely used cell lines for CHIKV antiviral drug screening. Fibroblast cell lines, such as MRC-5 (human lung fibroblast) or HFF-1 (human foreskin fibroblast), have also been used in multiple studies. Less frequently used cell lines include immortalized cells such as HeLa (human cervical carcinoma), Huh-7 or HepG2 (human hepatocellular carcinoma), and U2OS (human osteosarcoma). Cell lines usually vary with respect to drug uptake and intracellular metabolism. Therefore, it is anticipated that the compounds that first need to be metabolized into their active form, such as nucleoside analogues, might show cell line-dependent differences in their antiviral activity profiles. A study evaluating the anti-CHIKV efficacy of ribavirin and favipiravir, each of which needs to be converted to its active triphosphate form by host cell kinases, revealed differences in antiviral efficacy that depended on the cell line used for evaluation (119). Similarly, for immunomodulatory agents (not discussed in this review), the correct choice of cell line is especially relevant.

## CONCLUDING REMARKS

The lack of effective control measures and the spread of new vectors and increased human travel and urbanization greatly contributed to CHIKV reemergence between 2004 and 2020. The origin and scale of a future chikungunya outbreak are hard to predict, which underscores the importance of developing effective countermeasures. Identifying and developing direct-acting and host-targeting antiviral drug options against CHIKV infection offer a promising approach for limiting viral replication and spread.

The major complaint of patients suffering from CHIKF is debilitating joint and muscle pain, which results in lost productivity and reduced quality of life. Antiviral treatment would represent a suitable measure to prevent and treat CHIKV infections and significantly lower the burden of disease in affected areas. A combination therapy for CHIKF may prove useful to reduce the likelihood of developing drug resistance, given that compounds with different viral/host targets can produce synergistic effects. In addition, chronic CHIKF patients with exacerbated response of their immune system can also be treated with immunomodulatory agents to alleviate joint arthralgia and inflammation.

Validation of CHIKV small-molecule inhibitors is currently performed in a variety of *in vitro* and *in vivo* models. Ideally, *in vivo* antiviral testing is performed in animal models that replicate the clinical course of CHIKV infection in humans. While the use of an immunocompromised acute model, such as AG129 mice, may provide more-stringent conditions for antiviral evaluation, the use of an immunocompetent arthralgia model is more clinically relevant. The maximum beneficial effect of an antiviral compound for treatment of CHIKF patients would be achieved by early administration during the acute phase of infection, in order to reduce the viral load and decrease the likelihood of developing chronic manifestations. Clinical studies performed with patient material have already indicated that residual viral material (RNA/protein) in joint tissue, rather than replicating virus, likely contributes to the immunopathology that is associated with CHIKV infection (120). Consequently, late antiviral treatment, i.e., during the chronic phase of CHIKV infection, targeting specific CHIKV functions and host pathways involved in viral replication would be less effective given the absence or low quantities of full-length viral RNA. This stresses the importance of fully understanding the fundamental aspects of CHIKV-host interactions in patients with both acute and chronic disease. In summary, the development of CHIKV small-molecule inhibitors is justified for both prophylactic and therapeutic use. Given the current absence of a vaccine, availability of a clinically approved CHIKV small-molecule inhibitor would be especially advantageous in outbreak containment. Alternatively, it could be prescribed as a form of prophylaxis to local citizens in affected areas or to at-risk travelers.
